# Ginseng Gintonin Contains Ligands for GPR40 and GPR55

**DOI:** 10.3390/molecules25051102

**Published:** 2020-03-02

**Authors:** Yeon-Jin Cho, Sun-Hye Choi, Rami Lee, Hongik Hwang, Hyewhon Rhim, Ik-Hyun Cho, Hyoung-Chun Kim, Jeong-Ik Lee, Sung-Hee Hwang, Seung-Yeol Nah

**Affiliations:** 1 Ginsentology Research Laboratory and Department of Physiology, College of Veterinary Medicine, Konkuk University, Seoul 05029, Korea; yeonjin0202@naver.com (Y.-J.C.); vettman@naver.com (S.-H.C.); rmlee12@konkuk.ac.kr (R.L.); 2 Center for Neuroscience, Korea Institute of Science and Technology, Seoul 02792, Korea; hongik@kist.re.kr (H.H.); e-hrhim@kist.re.kr (H.R.); 3 Department of Convergence Medical Science, Department of Science in Korean Medicine and Brain Korea 21 Plus Program, Graduate School, Kyung Hee University, Seoul 02447, Korea; ihcho@khu.ac.kr; 4 Neuropsychopharmacology and Toxicology program, College of Pharmacy, Kangwon National University, Chunchon 24341, Korea; kimhc@kangwon.ac.kr; 5 Department of Veterinary Obstetrics and Theriogenology, College of Veterinary Medicine, Konkuk University, Seoul 05029, Korea; jeongik@konkuk.ac.kr; 6 Department of Pharmaceutical Engineering, College of Health Sciences, Sangji University, Wonju 26339, Korea

**Keywords:** ginseng, gintonin, GPR40, GPR55, insulin secretion, cell migration

## Abstract

Gintonin, a novel ginseng-derived glycolipoprotein complex, has an exogenous ligand for lysophosphatidic acid (LPA) receptors. However, recent lipid analysis of gintonin has shown that gintonin also contains other bioactive lipids besides LPAs, including linoleic acid and lysophosphatidylinositol (LPI). Linoleic acid, a free fatty acid, and LPI are known as ligands for the G-protein coupled receptors (GPCR), GPR40, and GPR55, respectively. We, herein, investigated whether gintonin could serve as a ligand for GPR40 and GPR55, using the insulin-secreting beta cell-derived cell line INS-1 and the human prostate cancer cell line PC-3, respectively. Gintonin dose-dependently enhanced insulin secretion from INS-1 cells. Gintonin-stimulated insulin secretion was partially inhibited by a GPR40 receptor antagonist but not an LPA1/3 receptor antagonist and was down-regulated by small interfering RNA (siRNA) against GPR40. Gintonin dose-dependently induced [Ca^2+^]_i_ transients and Ca^2+^-dependent cell migration in PC-3 cells. Gintonin actions in PC-3 cells were attenuated by pretreatment with a GPR55 antagonist and an LPA1/3 receptor antagonist or by down-regulating GPR55 with siRNA. Taken together, these results demonstrated that gintonin-mediated insulin secretion by INS-1 cells and PC-3 cell migration were regulated by the respective activation of GPR40 and GPR55 receptors. These findings indicated that gintonin could function as a ligand for both receptors. Finally, we demonstrated that gintonin contained two more GPCR ligands, in addition to that for LPA receptors. Gintonin, with its multiple GPCR ligands, might provide the molecular basis for the multiple pharmacological actions of ginseng.

## 1. Introduction

Ginseng, the root of *Panax ginseng* C.A. Meyer, has been used as a tonic in traditional medicine for many centuries [1,2]. The efforts of many scientists have revealed that ginseng has diverse pharmacological effects, including memory improvement, anti-tumor activity, immune system enhancement, anti-fatigue and anti-stress effects, and mitigation of metabolic disorders, such as diabetes [1,2]. Ginseng is thought to exert its diverse pharmacological effects via various active ingredients, including ginsenosides, acidic polysaccharides, and other minor anti-oxidative aromatic components [1,2]. 

Recently, we identified a novel ginseng component called gintonin [3,4]. Gintonin consists of carbohydrates, proteins, and lipids [3]. We later demonstrated that lysophosphatidic acids (LPAs) were a major functional component of gintonin [4] and showed that gintonin could activate LPA receptors, a kind of G-protein coupled receptor (GPCR), in animal cells. We reported that gintonin exerted diverse cellular effects in vitro through LPA receptor activation, including transient intracellular calcium mobilization, morphological changes, enhancement of proliferation and migration, vascular development, and neurite retraction [5,6,7,8,9]. Gintonin has also consistently shown memory improvement, hippocampal cell proliferation, and neurodegenerative disease antagonism in animal models [5,6,9,10,11].

More recently, lipid analysis of gintonin-enriched fractions (GEF) from ginseng has been qualitatively and quantitatively performed using gas chromatography-mass spectrometry and liquid chromatography-tandem mass spectrometry [12]. The results show that GEF contains fatty acids, such as linoleic acid (C18:2) (approximately 7.5%), palmitic acid (C16:0), and oleic acid (C18:1) [12]. GEF is also found to contain different phospholipids besides LPA (0.2% LPA C18:2, 0.06% LPA C16:0), such as lysophosphatidylinositol (LPI C18:2) (approximately 0.13%) and phosphatidic acid (PA) (1% PA 16:0–18:2, 0.5% PA 18:2–18:2) [12]. These findings indicate that GEF contains a relatively large amount of bioactive linoleic acid (C18:2), LPIs, and PAs, in addition to LPA C18:2. 

Linoleic acid is a fatty acid known to enhance insulin secretion from pancreatic beta cells through activation of the GPCR GPR40/free fatty acid receptor [13,14,15,16]. GPR40 is a potential therapeutic target in diabetes and may lead to the development of new medication [14]. Free fatty acid receptor GPR40 agonists, such as fasiglifam (TAK-875), have also shown efficacy in increasing insulin secretion in rat beta cells and lowering blood glucose [14,15,16]. LPI is a ligand for GPCR GPR55, which is also known as an endocannabinoid receptor [17,18,19,20]. Activation of GPR55 can trigger cell signaling cascades that stimulate cell proliferation and migration in certain cell types, such as transformed thyroid cells, lymphoblastoid cells, breast cancer cells, and prostate cancer cells [17,18,19,20,21]. In addition, GPR55 activation can regulate various physiological functions of the central nervous system [22,23,24].

As mentioned above, gintonin has been intensively studied as an LPA receptor-ligand source. However, a recent study has shown that pharmacological activities, such as stimulation of insulin secretion, are not dependent on LPA receptor activation [12]. Lipid analysis of GEF has shown relatively high amounts of linoleic acid and LPI [12] and has raised the possibility that GEF contains additional ligands for targets besides LPA receptors, such as GPR40 and GPR55. No previous reports have shown evidence that gintonin contains ligands for GPR40 and GPR55. Here, we investigated the effects of gintonin on insulin release in INS-1 rat pancreatic beta cells and on cell migration of PC-3 prostate cancer cells, to elucidate whether gintonin can also act on GPR40 and GPR55. We provided evidence that gintonin could act as a ligand for GPR40 and GPR55, using GPR40 and GPR55 antagonists, siRNA experiments, and signaling inhibitors. Finally, we discussed the physiological and pharmacological roles of gintonin through its ability to regulate multiple GPCRs, including GPR40 and GPR55, in biological systems.

## 2. Results

### 2.1. Gintonin-Induced Insulin Secretion in INS-1 Cells and Rat Islets

**Figure 1:**
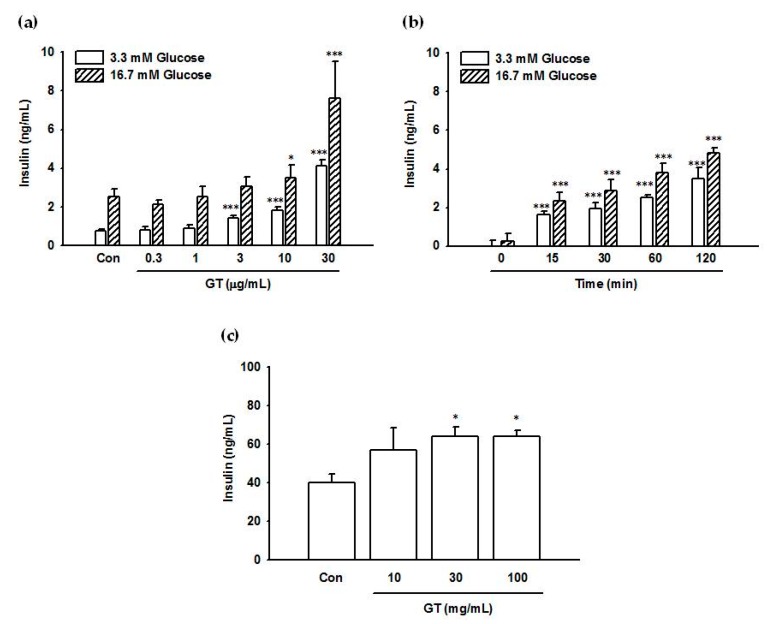
Effects of gintonin on insulin secretion from INS-1 cells and rat pancreatic islets. (**a**,**b**) Cells were preincubated in Krebs-Ringer bicarbonate (KRB) buffer (0.2 mM glucose) for 1 h before incubation with gintonin. (**a**) Cells were incubated with the indicated concentrations of gintonin (GT) in KRB buffer (3.3 mM glucose or 16.7 mM glucose) for 2 h. (**b**) Cells were incubated with gintonin (30 μg/mL) in KRB buffer (3.3 mM glucose or 16.7 mM glucose) for the indicated periods. (**c**) Rat pancreatic islets were preincubated with RPMI1640/F12 containing 5.6 mM glucose for 1 h and subsequently incubated with the indicated concentrations of gintonin in RPMI1640/F12 containing 5.6 mM glucose for another 1 h. Insulin secretion was measured using an insulin ELISA kit, as described in Materials and Methods. (**a**) Data represent means ± SD (*n* = 4); * *p* < 0.05, *** *p* < 0.001, vs. control (Con). (**b**) Data represent means ± SD (*n* = 3); *** *p* < 0.001, vs. time point 0 (quiescent cells). GT, gintonin. (**c**) Data represent means ± SD (*n* = 4 to 6); * *p* < 0.05, vs. control (Con). GT, gintonin.

Insulin secretion from INS-1 cells was examined after a 2 h exposure to 0–30 μg/mL gintonin in the presence of low (3.3 mM) and high (16.7 mM) glucose concentrations. As shown in Figure 1a, gintonin dose-dependently stimulated insulin secretion at both glucose concentrations. Dependence of insulin secretion on gintonin exposure time (0 to 2 h) is shown in Figure 1b. Gintonin (30–100 μg/mL) also stimulated insulin secretion from rat pancreatic islets in the presence of 5.6 mM glucose (Figure 1c). Gintonin treatment did not influence cell viability, indicating no cytotoxicity toward INS-1 cells at the indicated concentrations (Figure S1). Interestingly, gintonin did not increase transient intracellular calcium mobilization to modulate insulin secretion in INS-1 cells (data not shown).

### 2.2. Expression of GPR40 and LPA Receptors in INS-1 Cells

**Figure 2:**
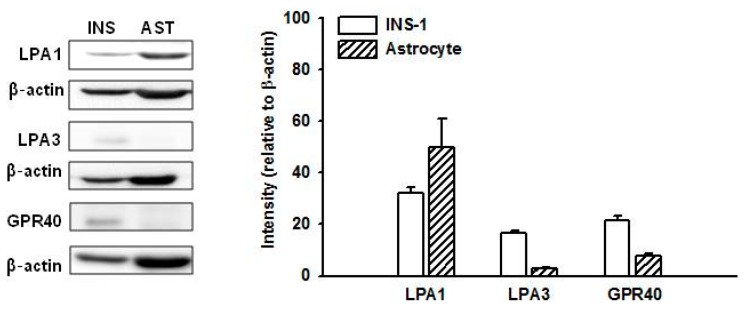
Expression of LPA1, LPA3, and GPR40 in INS-1 cells and mouse astrocytes. Cell lysates were subjected to immunoblotting using antibodies against LPA1, LPA3, and GPR40. The expression of each receptor relative to β-actin was plotted. Data represent means ± SD (*n* = 4). INS, INS-1 cells; AST, mouse astrocytes. LPA: lysophosphatidic acid.

It has recently been reported that GPR40, a free fatty acid receptor, may be involved in insulin secretion. It is thus a candidate therapeutic target for type 2 diabetes [14,15,16]. To confirm which receptors are involved in gintonin-stimulated insulin secretion, expression of GPR40 and representative LPA receptors was investigated by immunoblotting. As shown in Figure 2, GPR40 and LPA3 receptors were more strongly expressed in INS-1 cells than in mouse astrocytes, although LPA3 expression levels were very low. LPA1 receptors were weakly expressed in INS-1 cells compared to levels in mouse astrocytes, which are known to express LPA1 abundantly and LPA3 at relatively low levels [25,26].

### 2.3. Suppression of GPR40 Expression Reduced Gintonin-Induced Insulin Secretion from INS-1 Cells

**Figure 3:**
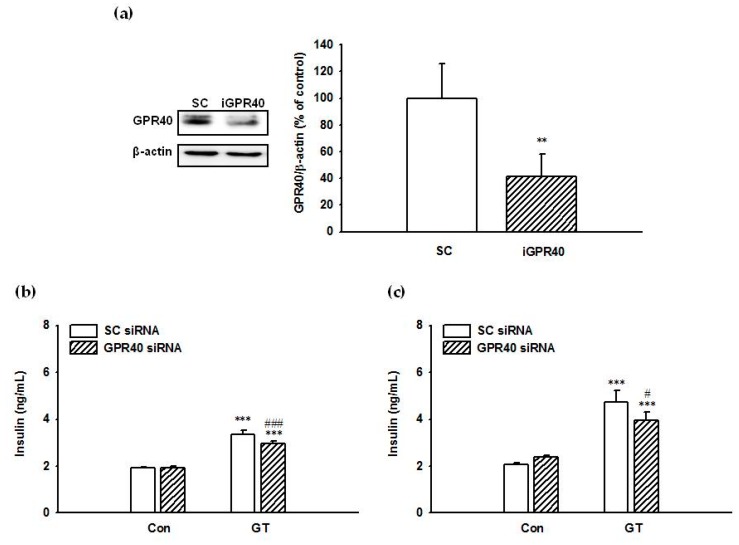
Effect of siRNA treatment on GPR40 expression (**a**) and gintonin-induced insulin secretion (**b**), (**c**) in INS-1 cells. INS-1 cells were transfected with either scrambled siRNA (SC) or GPR40 siRNA (iGPR40) and incubated for 3 days. (**a**) Immunoblot analysis of GPR40 levels in INS-1 cells transfected with either scrambled siRNA (SC) or GPR40 siRNA. Data represent means ± SD (*n* = 4); ** *p* < 0.01, vs. sc siRNA. (**b**), (**c**) Transfected INS-1 cells were incubated with or without gintonin (30 μg/mL) in KRB buffer (3.3 mM glucose (**b**) or 16.7 mM glucose (**c**)) for 2 h. Insulin secretion was measured using an insulin ELISA kit, as described in Materials and Methods. Data represent means ± SD (*n* = 4); ** *p* < 0.01, *** *p* < 0.001, vs. control. ^#^ *p* < 0.05, ^###^ *p* < 0.001, vs. sc siRNA. GT, gintonin.

We next examined the involvement of GPR40 in gintonin-induced insulin secretion. Transfection of INS-1 cells with GPR40 siRNA significantly decreased the expression of GPR40 protein by INS-1 cells compared to scrambled siRNA (negative control) transfected cells (Figure 3a). After 2 h incubation with 30 μg/mL of gintonin, insulin secretion from INS-1 cells transfected with scrambled siRNA was 3.35 ± 0.16 ng/mL and 4.73 ± 0.50 ng/mL in 3.3 mM (Figure 3b) and 16.7 mM (Figure 3c) glucose, respectively. After 2 h incubation with 30 μg/mL of gintonin, insulin secretion from INS-1 cells transfected with GPR40 siRNA was 2.96 ± 0.11 ng/mL and 3.96 ± 0.35 ng/mL in 3.3 mM (Figure 3b) and 16.7 mM (Figure 3c) glucose, respectively. Gintonin-induced insulin secretion was significantly decreased by GPR40 down-regulation by 11.6% and 16.3% at 3.3 mM glucose (Figure 3b) and 16.7 mM (Figure 3c), respectively. These data demonstrated that gintonin-stimulated insulin secretion from INS-1 cells was partially mediated by GPR40 activation.

### 2.4. GPR40 and Protein Kinase C (PKC) Inhibitors Attenuated Gintonin-Induced Insulin Secretion from INS-1 Cells

**Figure 4:**
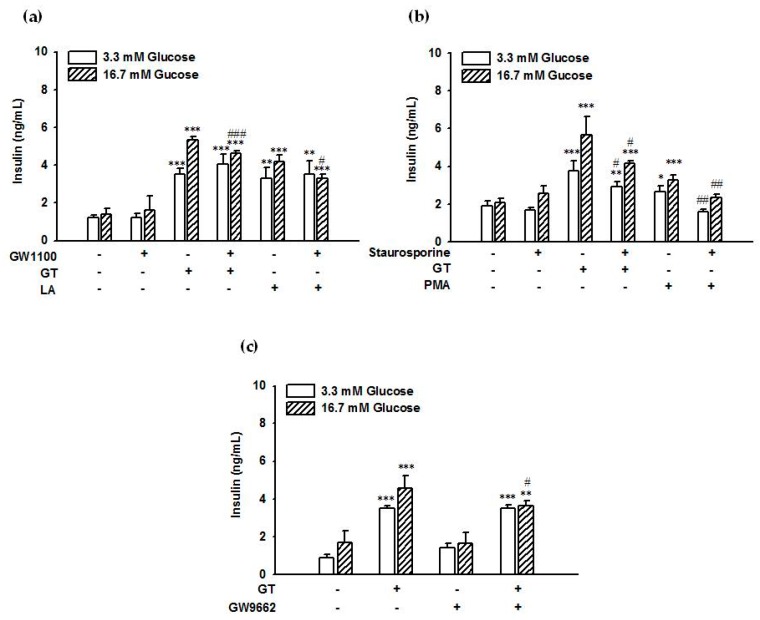
Inhibitory effects of GPR40 antagonist, PKC, and PPARγ inhibitors on gintonin-induced insulin secretion. Cells were incubated with gintonin (30 μg/mL), linoleic acid (200 μM, LA), or PMA (1 μM) in KRB buffer (3.3 mM glucose or 16.7 mM glucose) in the presence or absence of the GPR40 inhibitor GW1100 (5 μM) (**a**), the PKC inhibitor staurosporine (1 μM) (**b**), or the PPARγ inhibitor GW9662 (30 μM) (**c**) for 2 h. Various agents used here were dissolved in DMSO. Insulin secretion was measured using an insulin ELISA kit, as described in Materials and Methods. Data represent means ± SD (*n* = 4 to 6); * *p* < 0.05, ** *p* < 0.01, *** *p* < 0.001, vs. control (Con). ^#^ *p* < 0.05, ^##^ *p* < 0.01, ^###^ *p* < 0.001, vs. GT alone. GT, gintonin; LA, linoleic acid; PMA, phorbol 12-myristate 13-acetate; PKC, protein kinase C; PPARγ, peroxisome proliferator-activated receptor γ.

Gintonin (30 μg/mL) stimulated insulin secretion from INS-1 cells (3.52 ± 0.29 ng/mL and 5.34 ± 0.16 ng/mL) compared to control cells treated with vehicle (1.22 ± 0.16 ng/mL and 1.40 ± 0.30 ng/mL) in the presence of 3.3 mM or 16.7 mM glucose, respectively (Figure 4a). The GPR40 antagonist GW1100 (5 μM) attenuated gintonin-induced insulin secretion in the presence of 16.7 mM glucose (5.34 ± 0.16 ng/mL vs. 4.61 ± 0.14 ng/mL) but not 3.3 mM glucose (3.52 ± 0.29 ng/mL vs. 4.06 ± 0.53 ng/mL) (Figure 4a), indicating that GPR40 was involved in gintonin-stimulated insulin secretion from rat pancreatic cells. Linoleic acid (200 μM), as a positive control, also stimulated insulin secretion from INS-1 cells (3.32 ± 0.54 ng/mL and 4.21 ± 0.34 ng/mL) compared to control cells treated with vehicle in the presence of 3.3 mM or 16.7 mM glucose (Figure 4a). Stimulation by linoleic acid was also inhibited by GW1100 (5 μM) in the presence of 16.7 mM glucose (4.21 ± 0.34 ng/mL vs. 3.30 ± 0.21 ng/mL) but not 3.3 mM glucose (3.32 ± 0.54 ng/mL vs. 3.52 ± 0.73 ng/mL) (Figure 4a). The PKC receptor inhibitor staurosporine (1 μM) partially attenuated gintonin-stimulated insulin secretion in the presence of 3.3 mM glucose (3.76 ± 0.54 ng/mL vs. 2.93 ± 0.26 ng/mL) and 16.7 mM glucose (5.67 ± 0.99 ng/mL vs. 4.17 ± 0.12 ng/mL) (Figure 4c). Staurosporine (1 μM) also completely blocked phorbol 12-myristate 13-acetate (PMA)-stimulated insulin secretion in the presence of 3.3 mM glucose (2.66 ± 0.33 ng/mL vs. 1.60 ± 0.12 ng/mL) and 16.7 mM glucose (3.26 ± 0.28 ng/mL vs. 2.34 ± 0.17 ng/mL) (Figure 4b). The antagonist Ki16425, which targets LPA receptor subtypes LPA1/3 (LPA1/3), did not influence gintonin-induced insulin secretion (Figure S2a). Neither did the phospholipase C (PLC) inhibitor U73122 nor the calcium chelator 1,2-Bis(2-aminophenoxy)ethane-N,N,N′,N′-tetraacetic acid tetrakis(acetoxymethyl ester) (BAPTA-AM) (Figure S2b,c), indicating that gintonin did not induce insulin secretion via LPA receptor/PLC/Ca^2+^ signaling pathways. These results indicated that gintonin (30 μg/mL) stimulated insulin secretion from INS-1 cells through GPR40 activation and PKC activation, not through LPA receptor activation. On the other hand, although INS-1E cells, which are derived from INS-1 cells, did not express much GPR55 [27], we also examined whether GPR55 antagonist CID1261822 (ML193) (10 μM) could block gintonin-stimulated insulin release in INS-1 cells. We found that CID1261822 slightly but significantly inhibited gintonin-stimulated insulin release from INS-1 cells (Figure S2d).

### 2.5. The PPARγ Inhibitor Partially Attenuated Gintonin-Induced Insulin Secretion from INS-1 Cells

Gintonin-stimulated insulin secretion was also partially inhibited by the peroxisome proliferator-activated receptor (PPAR)γ inhibitor GW9662 (4.58 ± 0.64 ng/mL vs. 3.65 ± 0.27 ng/mL) in the presence of 16.7 mM glucose but not 3.3 mM glucose (Figure 4c). These results raised the possibility that gintonin-stimulated insulin secretion was achieved via multiple signaling pathways.

### 2.6. Effect of Gintonin on Intracellular Calcium Mobilization in PC-3 Cells

**Figure 5:**
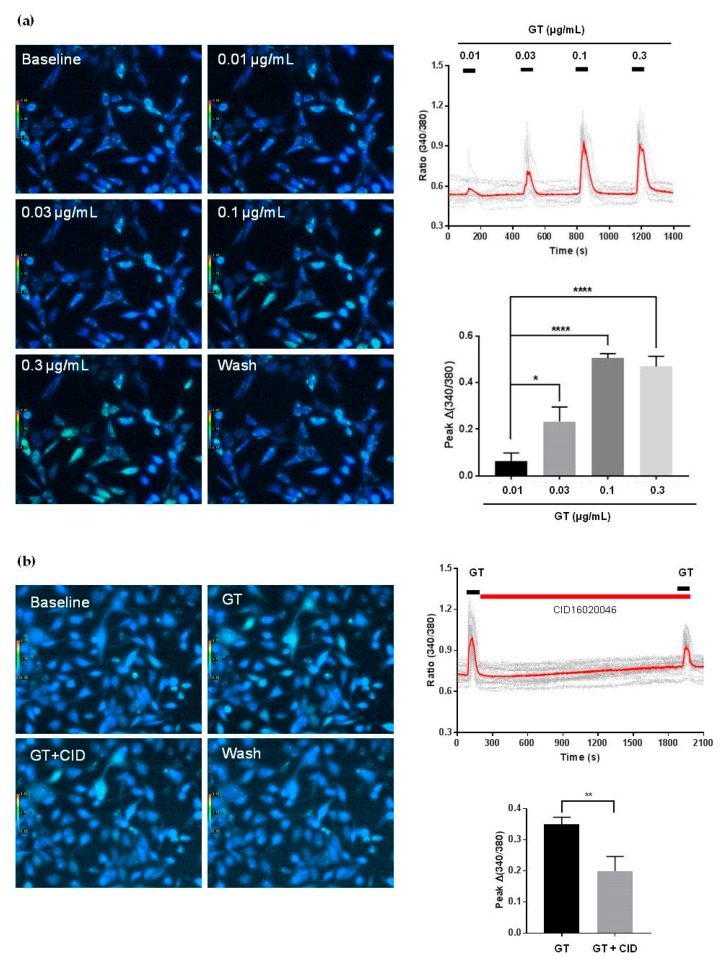
Effects of gintonin on transients [Ca^2+^]_i_ (**a**), and effects of the GPR55 antagonist CID16020046 on gintonin (0.1 μg/mL)-mediated transients [Ca^2+^]_i_ (**b**) in PC-3 cells. Analyses of the localization of calcium signals via confocal microscopy were performed with Fura-2/AM and a nuclear dye. (**a**) Intracellular Ca^2+^ levels were determined in PC-3 cells exposed to gintonin (0.01, 0.03, 0.1, and 0.3 μg/mL). PC-3 cells from each group were incubated for 40–60 min at room temperature with 5 μM Fura-2/AM (Thermo Fisher Scientific Korea, Gangnam-gu, Seoul, Korea) and 0.001% Pluronic F-127 (Thermo Fisher Scientific Korea, Gangnam-gu, Seoul, Korea) in a HEPES-buffered solution (pH 7.4). Cells were illuminated using a xenon arc lamp, and excitation wavelengths (340 and 380 nm) were selected using a computer-controlled filter wheel (Sutter Instrument, Novato, CA, USA). Left panel: representative pictures of [Ca^2+^]_i_ levels in PC-3 cells in the absence or presence of various concentrations of gintonin. Upper right panel: representative [Ca^2+^]_i_ peaks associated with different gintonin concentrations. Lower right panel: histograms of the dose-dependent responses to gintonin. (**b**) Left panel: representative pictures of [Ca^2+^]_i_ levels in PC-3 cells in the absence or presence of CID16020046 (10 μM). Upper right panel: representative peaks of [Ca^2+^]_i_ transients in the absence or presence of CID16020046. Lower right panel: histograms of the effects of CID16020046 on gintonin (0.1 μg/mL)-mediated [Ca^2+^]_I_ transients. Data were obtained from 45–50 different cells in three independent experiments. Data represent means ± SD. * *p* < 0.05, **** *p* < 0.0001 compared to 0.01 μg/mL gintonin (**a**); ** *p* < 0.01, compared to the 2nd GT application (**b**).

**Figure 6:**
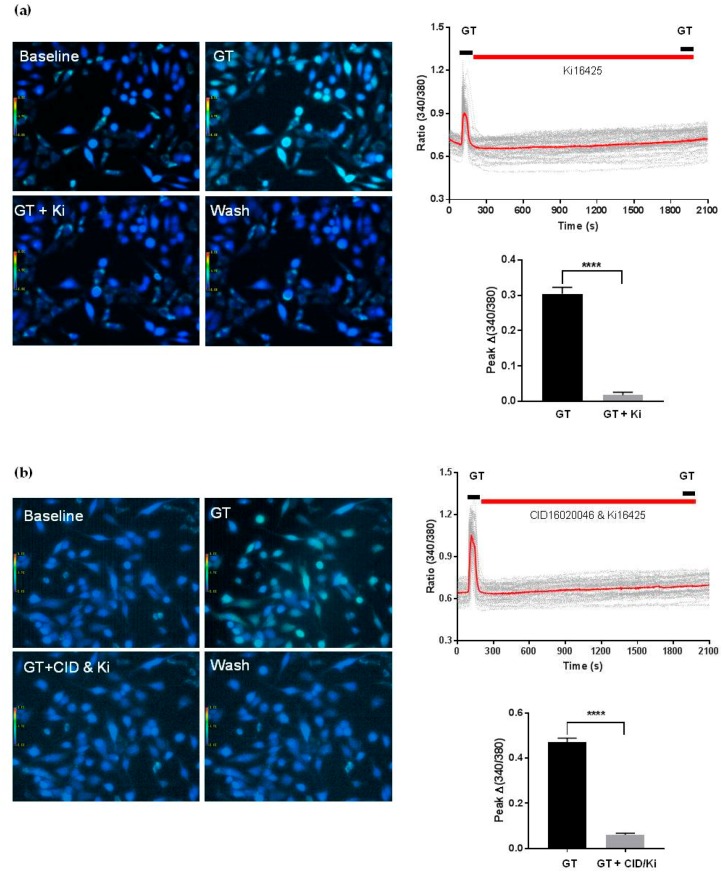
Effects of the LPA1/3 antagonist Ki16425 (**a**) alone and co-treatment with Ki16425 and the GPR55 antagonist CID16020046 (**b**) on gintonin (0.1 μg/mL)-mediated [Ca^2+^]_i_ transients in PC-3 cells. Analyses of calcium signal localization were performed, as described in Figure 5. Left panel (**a**,**b**): representative pictures of [Ca^2+^]_i_ levels in PC-3 cells in the absence or presence of Ki16425 (10 μM) or CID16020046 (10 μM) plus Ki16425. Upper right panel: representative peaks of [Ca^2+^]_i_ transients in the absence or presence of Ki16425 or CID16020046 (10 μM) plus Ki16425. Lower right panel (**a**,**b**): histograms of the effects of Ki16425 or CID16020046 (10 μM) plus Ki16425 on gintonin (0.1 μg/mL)-mediated [Ca^2+^]_i_ transients. Data were obtained from 40–50 different cells in three independent experiments. Data represent means ± SD. **** *p* < 0.0001, compared to the presence of inhibitor (Ki16425, Ki, or CID16020046 plus Ki). GT, gintonin.

Gintonin stimulated [Ca^2+^]_i_ mobilization in a dose-dependent manner at concentrations of 0.01–0.1 μg/mL. Stimulation reached a plateau at 0.1 μg/mL (Figure 5a). Gintonin-induced [Ca^2+^]_i_ mobilization was significantly reduced after treatment with the GPR55 antagonist CID16020046 (10 μM) (Figure 5b). Gintonin-induced [Ca^2+^]_i_ mobilization was also almost completely blocked after co-treatment with the LPA1/3 antagonist Ki16425 (10 μM) and the GPR55 antagonist CID16020046 (Figure 6a,b). These results indicated that PC-3 cells expressed both LPA1/3 and GPR55.

### 2.7. Effect of Gintonin on PC-3 Cell Migration

**Figure 7:**
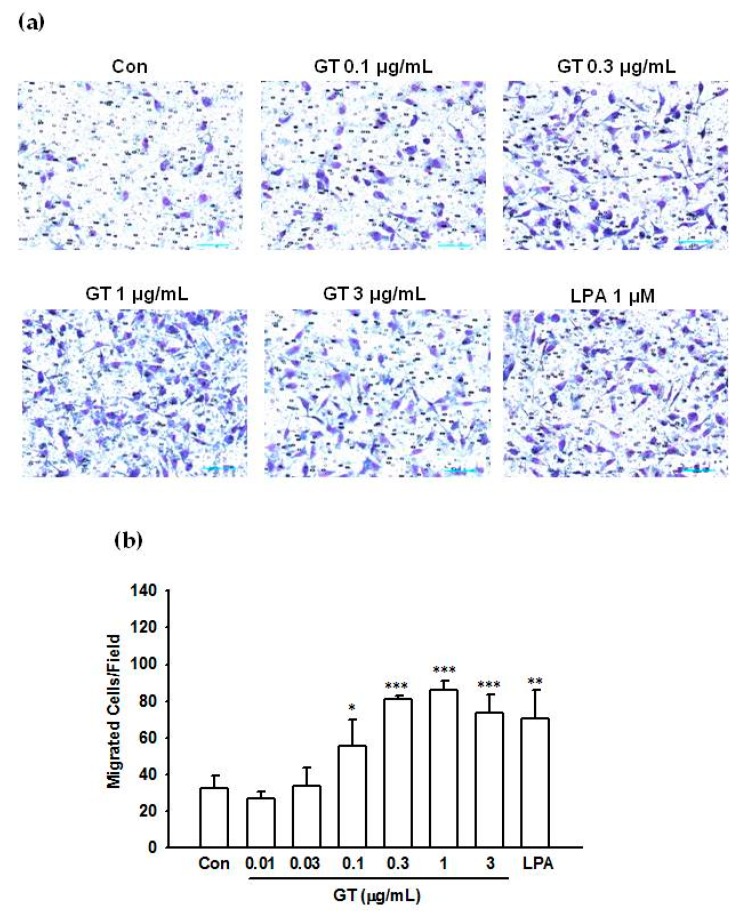
Effects of gintonin on PC-3 cell migration. Chemotactic migration was quantitated using modified Boyden chambers, as described in Materials and Methods. Cell migration of PC-3 cells treated with gintonin (0.01–3 μg/mL) was examined. Data were obtained from 4 fields per well, 4 different wells. (**a**) representative pictures of PC-3 cell migration. (**b**) histograms of the effects of gintonin on migration. Data represent means ± SD. * *p* < 0.05, ** *p* < 0.01, *** *p* < 0.001 compared to control (Con). Con, control; GT, gintonin.

Next, we examined the effect of gintonin on chemotactic motility using modified Boyden chambers, since LPI and LPA also stimulate PC-3 cell migration [21,28,29]. Gintonin stimulated PC-3 cell migration in a concentration-dependent manner at concentrations of 0.01–1 μg/mL. The response saturated at ≥1 μg/mL (Figure 7). Gintonin (0.3–3 μg/mL) and LPA (1 μM) only slightly stimulated cell proliferation after 24 h incubation (Figure S3). 

### 2.8. Gintonin-Mediated PC-3 Cell Migration Was Partially Inhibited by a GPR55 Inhibitor, an LPA1/3 Antagonist, and a Calcium Chelator

**Figure 8:**
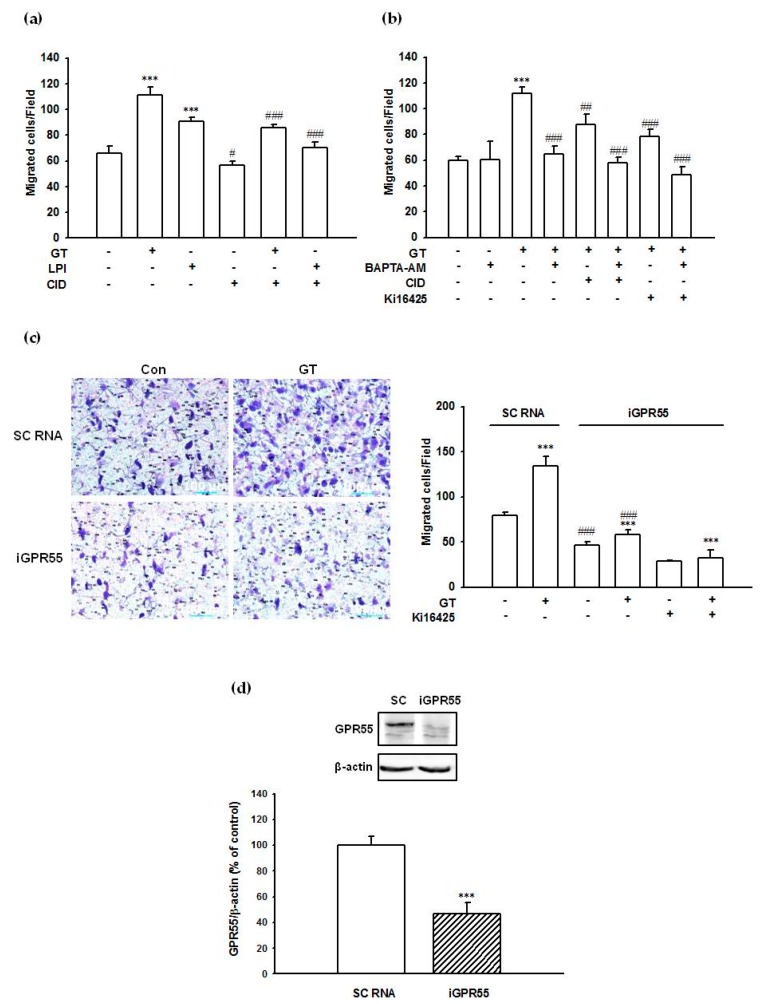
Inhibitory effects of a GPR55 antagonist, a calcium chelator, an LPA1/3 antagonist, and GPR55 silencing via siRNA on gintonin-induced migration and effect of siRNA treatment on GPR55 expression. Cells were incubated with gintonin (1 μg/mL) or LPI (1 μM) in serum-free RPMI1640 medium in the presence or absence of the GPR55 antagonist CID16020046 (10 μM) (**a**,**b**), the calcium chelator BAPTA (5 μM) (**b**), or the LPA1/3 antagonist Ki16425 (10 μM) (**b**,**c**) for 5 h. The migration of cells towards the gintonin-containing chamber through the polycarbonate membrane of modified Boyden chambers was quantitated, as described in Materials and Methods (**a**–**c**). Data represent means ± SD (*n* = 4); *** *p* < 0.001, vs. control (Con). ^#^ *p* < 0.05, ^##^ *p* < 0.01, ^###^ *p* < 0.001, vs. GT alone. GT, gintonin; LPI, lysophosphatidylinositol; CID, CID16020046 (**a**–**c**). (**d**) PC-3 cells were transfected with either scrambled siRNA (SC) or GPR55 siRNA (iGPR40) and incubated for 2 days. Immunoblot analysis of GPR55 levels in PC-3 cells that had been transfected with either scrambled siRNA (SC) or GPR55 siRNA. Data represent means ± SD (*n* = 4); *** *p* < 0.001, vs. sc siRNA.

Treatment with 1 μg/mL gintonin stimulated PC-3 cell migration by approximately 69–86% compared to that of control cells (Figure 8a–c). LPI (1 μM) stimulated PC-3 migration by 38% compared to that of control cells (Figure 8a). The GPR55 antagonist CID16020046 (10 μM) inhibited gintonin- and LPI-stimulated PC-3 cell migration by approximately 56% and 82%, respectively (Figure 8a). The LPA1/3 antagonist Ki16425 (10 μM) also inhibited gintonin-stimulated PC-3 cell migration by approximately 65% (Figure 8b). The calcium chelator BAPTA-AM (5 μM) also inhibited gintonin-stimulated PC-3 cell migration by approximately 91% (Figure 8b). Co-treatment with CID16020046 (10 μM) or Ki16425 (10 μM) and BAPTA-AM (5 μM) completely blocked gintonin-stimulated PC-3 cell migration (Figure 8b). These results indicated that both LPAs and LPIs in gintonin stimulated cell migration via both LPA1/3 receptor- and GPR55-mediated Ca^2+^ signaling pathways.

### 2.9. Suppression of GPR55 Expression by siRNA Reduced Gintonin-Mediated PC-3 Cell Migration

To examine the involvement of GPR55 in gintonin-mediated PC-3 cell migration, GPR55 expression was suppressed by siRNA transfection. Transfection of PC-3 cells with GPR55 siRNA significantly decreased cell migration (by about 41%) and GPR55 protein expression compared to scrambled siRNA (negative control) transfected cells (by about 53%) (Figure 8d). Gintonin (1 μg/mL) stimulated cell migration by 70% and 24% in PC-3 cells transfected with scrambled siRNA and with siRNA against GPR55, respectively, indicating that downregulation of GPR55 reduced gintonin-stimulated PC-3 cell migration (Figure 8c). Gintonin-stimulated migration of PC-3 cells transfected with siRNA against GPR55 was significantly decreased (51%) by treatment with Ki16425 (Figure 8c), indicating the involvement of LPA1/3 and GPR55 receptors in gintonin-stimulated migration in addition to GPR55.

## 3. Discussion

In previous reports, we have shown that gintonin is a ginseng-derived glycolipoprotein complex. Its main functional ingredients are LPAs. We have also shown that gintonin LPAs function as an LPA receptor-ligand exhibiting diverse biological effects in vitro and in vivo [4,5,6,7,8,9]. In a subsequent study, we showed that gintonin contains other bioactive lipids besides LPAs, listed in order of prevalence: linoleic acid > phosphatidic acids > LPAs > LPIs [12]. However, prior to this study, it was not known whether linoleic acid and LPI in gintonin could exhibit physiological effects as ligands of other receptors. In the present study, we provided evidence that linoleic acid and LPI in gintonin could act as a ligand of GPR40 and GPR55 through insulin secretion and cell migration assays, respectively.

We first examined whether linoleic acid in gintonin could act on GPR40, a known free fatty acid receptor. Free fatty acids, such as linoleic acid, have been known to stimulate insulin secretion by activating the fatty acid receptor GPR40/free fatty acid receptor 1 (FFAR1), which is expressed in pancreatic β cells [30,31]. GPR40 agonists have been known to stimulate insulin secretion and lower glucose levels and can be used as antidiabetic drugs [32]. In the present study, we observed that gintonin dose- and time-dependently stimulated insulin secretion by INS-1 cells in the presence of 3.3 mM and 16.7 mM glucose (Figure 1a,b). Immunoblotting analysis showed that GPR40 was expressed in INS-1 rat insulinoma cells, consistent with previous reports [33] (Figure 2). Transfection of INS-1 cells with siRNA against GPR40 resulted in GPR40 down-regulation (Figure 3a) and partially reduced insulin secretion compared to the cells transfected with scrambled siRNA (Figure 3b,c). Co-treatment with the GPR40 antagonist, GW1100, also partially inhibited gintonin-stimulated insulin secretion (Figure 4a). Gintonin-induced insulin secretion was inhibited by GW1100 only under hyperglycemic conditions (16.7 mM glucose), indicating that gintonin-mediated augmentation of insulin secretion was glucose-dependent, as previously reported for long-chain fatty acid-stimulation of insulin secretion via GPR40 [7]. However, the LPA 1/3 antagonist Ki16425 did not influence gintonin-mediated insulin secretion (Figure S2a), consistent with a previous report on GEF [12].

**Figure 9:**
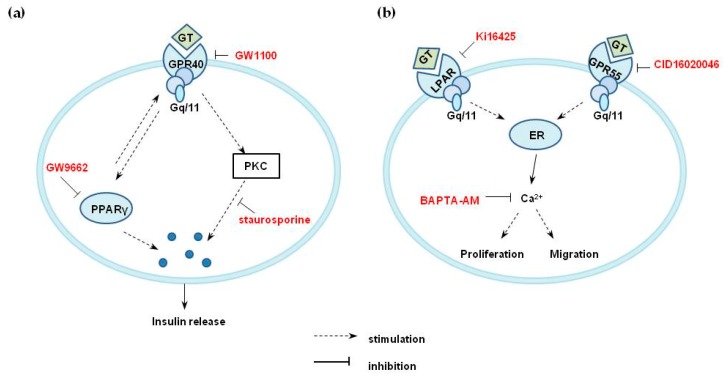
Proposed mechanisms for gintonin-induced insulin secretion in INS-1 cells (**a**), and gintonin-induced cell migration in PC-3 cells (**b**). (**a**) GPR40 activation by gintonin (GT) stimulated insulin secretion via PKC activation or other unknown signaling pathways in INS-1 cells. Neither PLC activation nor intracellular calcium mobilization was seen with the gintonin treatment of INS-1 cells. PPARγ activation by gintonin might be partially responsible for gintonin-induced insulin secretion through indirect pathways. (**b**) GPR55 activation by gintonin (GT) might stimulate calcium release from the endoplasmic reticulum, elicit intracellular calcium mobilization, and induce cell migration in PC-3 cells. GT, gintonin; ER, endoplasmic reticulum.

Signaling pathways downstream of G protein-coupled receptors that activate insulin secretion by pancreatic β-cells might include diverse pathways, such as Gα_s_-cAMP-PKA, Gα_q/11_-PLC-PKC, and/or intracellular Ca^2+^ [34]. Increased cAMP is reported in insulin secretion mediated by GPR119, glucagon-like peptide 1, glucose-dependent insulinotropic peptide, or GPR120 activation. However, gintonin did not increase the cAMP level in INS-1 cells (data not shown). A panel of saturated and mono- or polyunsaturated fatty acids act as GPR40 agonists and induces [Ca^2+^]_i_ transient through phospholipase C (PLC)-signaling pathway [30,31]. Interestingly, in the present study, neither the PLC inhibitor U73122 nor the intracellular calcium chelator BAPTA-AM attenuated gintonin-induced insulin secretion in INS-1 cells, suggesting the possible involvement of mechanisms other than the PLC-calcium mobilization signaling pathway (Figure S2b). The PKC inhibitor staurosporine attenuated gintonin-stimulated insulin secretion, indicating the involvement of PKC pathways (Figure 4b). In this study, the treatment of INS-1 cells with the PPARγ inhibitor GW9662 partially reduced gintonin-induced insulin secretion under hyperglycemic conditions (16.7 mM glucose) (Figure 4c). Thus, it could be suggested that PPARγ activation partially stimulated a GPR40-mediated pathway of gintonin-induced insulin secretion (Figure 9a). GPR40, PKC, and PPARγ inhibitors all partially reduced gintonin-stimulated insulin secretion. Thus, it is likely that PPARγ activation was also involved in gintonin-mediated insulin secretion, especially under hyperglycemic conditions, similar to GPR40 activation. Further studies are required to clarify the detailed mechanisms. Taken together, these findings indicated that gintonin induced insulin secretion in pancreatic β cells via GPR40 signaling pathways, as shown in Figure 9a.

GPR55 is another GPCR, known as an LPI receptor and a putative endocannabinoid receptor, and is a peripheral target for diabetes treatment because GPR55 agonists have insulinotropic activity [22,23,24,34]. GPR55 is also reportedly a regulator of the migration of several types of cancer cells [21]. GPR55 is also involved in the regulation of osteoclast number and function and inflammatory and neuropathic pain [21]. LPI is known to be a ligand for GPR55 and can trigger intracellular calcium mobilization, cell growth, differentiation, and motility in certain cell types, similar to the functions of LPA and LPA receptors, although some effects of LPI are not mediated by GPR55 activation but by unknown signaling pathways [17,18,19,20,21]. LPI-mediated intracellular calcium mobilization and migration have been observed in PC-3 cells, which abundantly express the receptor, and the migratory effects of LPI on these cells are well explained [19,21,28]. In the present study, since gintonin also contains LPIs, we examined gintonin as an activator of GPR55 in PC-3 cells. Our results demonstrated that gintonin stimulated intracellular calcium mobilization, which was blocked by pretreatment with a GPR55 antagonist or with a calcium chelator. Gintonin-mediated chemotactic migration of PC-3 cells was significantly inhibited by pretreatment of cells with a GPR55 antagonist or a calcium chelator. Down-regulation of GPR55 by siRNA transfection also reduced the migration of PC-3 cells. These results indicated that gintonin could stimulate PC-3 cell migration through a GPR55 receptor-Ca^2+^ signaling pathway (Figure 9b).

Recent studies have shown that LPI is a potent endogenous GPR55 agonist that is found in the brain [22,23,24]. LPI and GPR55 are implicated as potential key modulators of stress responses, depression, motor functions, and memory in the central nervous system, since GPR55 is highly expressed in the cortex, hippocampus, striatum, and spinal cord [22,23,24]. In previous studies, we showed that gintonin attenuated depressive behaviors, enhanced motor function performance, and enhanced hippocampal-dependent cognitive functions [11,35,36]. Although we did not demonstrate that these gintonin-mediated effects on the central nervous system were directly achieved via GPR55, gintonin LPIs and LPAs might play important roles in various brain functions. Further studies are required to elucidate the role of LPIs in gintonin for brain functions.

In summary, using INS-1 cells, which express GPR40, and PC-3 cells, which express GPR55, we demonstrated that gintonin regulated insulin secretion and cell migration via the respective receptor activations. The present study showed that gintonin contained activating ligands for GPR40 and GPR55 in addition to LPA receptors. Thus, previous and present studies show that gintonin contains at least three different GPCR ligands: linoleic acid, LPAs, and LPIs. In conclusion, ginseng gintonin, with its multiple GPCR ligands, might provide the molecular basis for the multiple pharmacological actions of ginseng.

## 4. Materials and Methods 

### 4.1. Materials

Crude gintonin was isolated from *Panax ginseng,* as described in a previous report [3]. The main component of gintonin is a complex of lysophosphatidic acid and ginseng proteins [3]. An enzyme-linked immunosorbent assay (ELISA) kit for insulin was purchased from Shibayagi Co. Ltd. (Shibukawa, Gunma, Japan). 1-oleoyl-2-hydroxy-sn-glycero-3-phosphate (LPA C18:1) was purchased from Avanti Polar Lipids (Alabaster, AL, USA). GW-1100 was purchased from MedChem Express (Princeton, NJ, USA). Endothelial differentiation gene (EDG)-2 (LPA1) monoclonal antibody, EDG-7 (LPA3) monoclonal antibody, and goat anti-beta actin monoclonal antibody conjugated with horseradish peroxidase were purchased from Santa Cruz Biotechnology, Inc. (Dallas, TX, USA). The anti-FFAR1/GPR40 polyclonal antibody was purchased from NOVUS Biologicals (Littleton, CO, USA). Anti GPR55 polyclonal antibody and Ki16425 were purchased from Cayman Chemical (Ann Arbor, MI, USA). All siRNAs were purchased from Bioneer Corporation (Daejeon, South Korea). RPMI1640 medium and all other materials for cell culture were purchased from Thermo Fisher Scientific Korea (Gangnam-gu, Seoul, Korea). All other reagents used were purchased from Sigma-Aldrich (St. Louis, MO, USA).

### 4.2. Cell Culture

The INS-1 rat beta-cell line [37] was kindly provided by Prof. HS Jeon (Gachon University, Lee Gil Ya Cancer and Diabetes Institute, Incheon City, South Korea), and PC-3 cells [38] were kindly provided by Prof. JH Park (Seoul National University, College of Veterinary Medicine). INS-1 cells were cultured in RPMI1640 supplemented with 10% (*v/v*) fetal bovine serum (FBS), 55 μM β-mercaptoethanol, 100 units/mL penicillin, and 100 μg/mL streptomycin. PC-3 cells were cultured in RPMI1640 supplemented with 10 % (*v/v*) fetal bovine serum (FBS), 100 units/mL penicillin, and 100 μg/mL streptomycin. 

### 4.3. Cell Viability

Viability of INS-1 cells was determined by a sodium 2,3,-bis(2-methoxy-4-nitro-5-sulfophenyl)-5-[(phenylamino)-carbonyl]-2H-tetrazolium inner salt) (XTT)-based assay, as previously described, with some modifications [4,8]. Briefly, cells were seeded at 3 × 10^3^ cells per well into 96-well plates. After 24 h, cells were washed with serum-free RPMI1640. Cells were then washed with the fresh serum-free medium again and incubated with gintonin at the indicated concentrations. After 2 h or 48 h incubation, the culture medium was replaced with 100 μL of serum-free medium without phenol red. Twenty-five microliters of XTT reaction solution containing 1 mg/mL of XTT and 0.036 mg/mL of phenazine methylsulfate were added to each well. After 2 h incubation, absorbance was measured at 450 nm.

### 4.4. Insulin Secretion from INS-1 Cells

Insulin secretion from INS-1 cells was quantitated using an enzyme-linked immunosorbent assay (ELISA), as previously described [12]. Briefly, INS-1 cells were seeded at 3 × 10^5^ cells per well into 24-well plates. After 24 h, cells were washed with Krebs-Ringer bicarbonate (KRB) buffer (24 mM NaHCO_3_, 1.2 mM MgCl_2_, 1 mM HEPES, 129 mM NaCl, 4.8 mM KCl, 1.2 mM KH_2_PO_4_, 2.5 mM CaCl_2_, 0.2 mM glucose, pH 7.4) and then preincubated in KRB buffer for 1 h. Cells were then incubated with gintonin, LPA, linoleic acid, or phorbol 12-myristate 13-acetate (PMA; 12-O-Tetradecanoylphorbol-13-acetate; TPA) at indicated concentrations for 2 h. In some experiments, inhibitors were also added prior to gintonin, linoleic acid, or PMA treatment. Supernatants were collected, and the insulin concentration of each sample was determined using a rat insulin ELISA kit according to the manufacturer’s instructions (Shibayagi, Gunma, Japan).

### 4.5. Islet Isolation and Insulin Secretion Assay

Animal experiments were conducted in accordance with recommendations in the Guide for the Care and Use of Laboratory Animals of the National Institutes of Health. The protocol was approved by the Committee on the Ethics of Animal Experiments of Konkuk University (Permit Number: KU13003). Islets of Langerhans were isolated from the pancreas of male Sprague-Dawley rats, as previously described [39]. Islets (50 islets/well) were loaded onto insert wells in 12-well transwell plates (Costar 3402). Islets were preincubated for 1 h in complete RPMI1640/F12 (RPMI1640/F12 (1:1), 10% FBS, 0.3 mM ascorbic acid, 25 mM HEPES, 1% antibiotics, and antimycotics (ThermoFisher Scientific Korea, Gangnam-gu, Seoul, South Korea), with 5.6 mM glucose to equilibrate the islets.

### 4.6. Small-Interfering RNA Transfection

INS-1 cells were transfected with three siRNAs specific for rat GPR40, or with a scrambled siRNA as a negative control. The siRNA sequences for rat GPR40 were 5′-GUG UGG UAC UCA ACC CAC U-3′, 5′-ACA UAC CCG UGA AUG GCU C-3′, and 5′-CGA GGA CUC AAA GAG GAA C-3′ (Bioneer Corporation, Daejeon, South Korea). PC-3 cells were transfected with siRNAs for human GPR55 or with scrambled siRNA. The siRNA sequence for human GPR55 was 5′-AGG UGU UUG GCU UCC UCC UCC CCA U-3′ (Bioneer Corporation, Daejeon, South Korea). Transfection of siRNA at a final concentration of 100 nM was performed under serum-free conditions with Lipofectamine 2000 (ThermoFisher Scientific Korea, Gangnam-gu, Seoul, South Korea) according to the manufacturer’s instructions. After 5 h transfection, the transfection solution was replaced with the growth medium. Three or four days after transfection, receptor expression, insulin secretion, and cell migration were determined.

### 4.7. Western Blot Analysis 

Cells were lysed with modified RIPA buffer, and LPA receptors, GPR40, and GPR55 expression were detected by sodium dodecyl sulfate-polyacrylamide gel electrophoresis (SDS-PAGE), followed by immunoblotting using rabbit anti-EDG2/LPA1 polyclonal antibody (Abcam, Cambridge, UK), rabbit anti-EDG7/LPA3 polyclonal antibody (Abcam, Cambridge, UK), anti-FFAR1/GPR40 polyclonal antibody (NOVUS Biologicals, Littleton, CO, USA), or anti-GPR55 polyclonal antibody (Cayman Chemical, Ann Arbor, MI, USA). Probed membranes were then stripped and re-probed with mouse anti-β actin monoclonal antibody conjugated with HRP (Abcam, Cambridge, UK). For the detection of extracellular signaling-regulated kinase (ERK) phosphorylation, cells were stimulated with gintonin for 10 min and lysed with modified RIPA buffer. Then, ERK phosphorylation was determined by SDS-PAGE and immunoblotting with a rabbit anti-phospho-ERK polyclonal antibody (Cell signaling, Danvers, MA, USA), as previously described [4]. The probed membrane was then stripped and reprobed for total ERK with a rabbit anti-ERK polyclonal antibody (Cell signaling, Danvers, MA, USA). Data capture and processing were performed with a luminescent image analyzer LAS-4000 and Multi Gauge software (Fujifilm, Tokyo, Japan).

### 4.8. Migration Assay Using Modified Boyden Chambers

The chemotactic motility of PC-3 cells was measured using modified Boyden chambers (Neuro Probe, Gaithersburg, MD, USA), as previously described [4]. Briefly, polycarbonate membranes with 8 μm pore size (Neuro Probe, Gaithersburg, MD, USA) were coated with 0.1 mg/mL of collagen type I from rat tails (BD Bioscience, San Jose, CA, USA). Gintonin, LPA, or LPI in serum-free RPMI1640 was added to the lower chambers. The Boyden chambers were assembled by placing the membranes and upper chambers into the lower chambers. Cells (4 × 10^4^ cells/well) were loaded into the upper chambers and incubated for 5 h at 37 °C. In some experiments, cells were pretreated with or without inhibitors, then gintonin or LPI in RPMI1640 was added to the upper chambers, followed by incubation for an additional 4 h. Cells on the membrane were fixed and stained with Diff Quik (Sysmex, Kobe, Japan). Migrated cells in four randomly chosen fields per well (16 fields per group) were counted under a microscope (light microscopy) at a magnification of ×200. Images were photographed using dark field microscopy (Eclipse 80i; Nikon, Tokyo, Japan).

### 4.9. Measurement of Intracellular Calcium Concentrations 

Intracellular free calcium levels were measured by dual excitation spectrofluorometric analysis of cells loaded with Fura-2 AM (Ex: 340 nm and 380 nm, Em: 515 nm) and a nuclear dye (DAPI) via confocal microscopy after PC-3 cells were treated with gintonin, as previously described [10]. Briefly, intracellular free calcium levels of cells were assayed in HEPES-buffered saline solution (HBS, 150 mM NaCl, 5 mM KCl, 1 mM MgCl_2_, 2 mM CaCl_2_, 10 mM HEPES, and 10 mM glucose, pH 7.4). All images were reflected to a frame transfer-cooled CCD camera (Olympus, Japan), and ratios of emitted fluorescence at excitation wavelengths of 340 and 380 nm were calculated using a digital fluorescence analyzer; thereafter, the intracellular free Ca^2+^ concentrations [Ca^2+^]_i_ were calculated. All imaging data were collected and analyzed using MetaFluor software (Univeral Imaging Corp. Downing, PA, USA).

### 4.10. Statistical Analysis

Data are expressed as means ± standard deviation. Statistical comparisons of controls and treated experimental groups were performed using Student’s t-test. All statistical analyses were performed using GraphPad Prism, version 5.0 (Graph Pad Software). p-values less than 0.05 were considered statistically significant.

## Supplementary Materials

Figure S1: Effect of gintonin on cell viability in INS-1 cells, Figure S2: Effect of LPA1/3 receptor inhibitor, PLC inhibitor, calcium chelator, or GPR55 antagonist on gintonin-induced insulin secretion from INS-1 cells, Figure S3: Effect of gintonin on PC-3 cell viability, Figure S4: The stimulatory effect of gintonin on ERK phosphorylation in INS-1 cells.
