# Supplementary material for: Ginseng Gintonin Contains Ligands for GPR40 and GPR55
Source: Molecules. 2020 Mar 2;25(5):1102. doi: 10.3390/molecules25051102 (PMC7179172; doi:10.3390/molecules25051102)
Supplement: Supplementary file 1 [file molecules-25-01102-s001.zip › molecules-701912-SI.docx]

Supplementary Materials

Gintonin contains ligands for GPR40 and GPR55


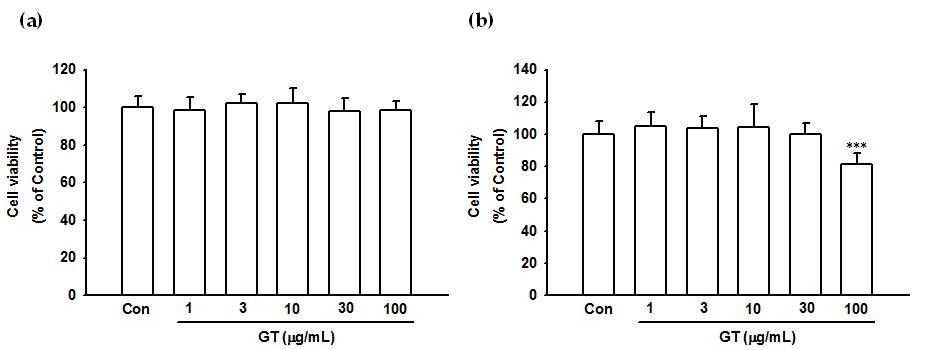


**Figure 1*.*** Effect of gintonin on cell viability in INS-1 cells. Cells were incubated with the indicated concentrations of gintonin for 3h (a) or 48 h (b). Cell viability was examined by XTT-based cell viability assay. Data represent means ± SD (n=4 to 6); ***p < 0.001, vs. control (Con). GT, gintonin.


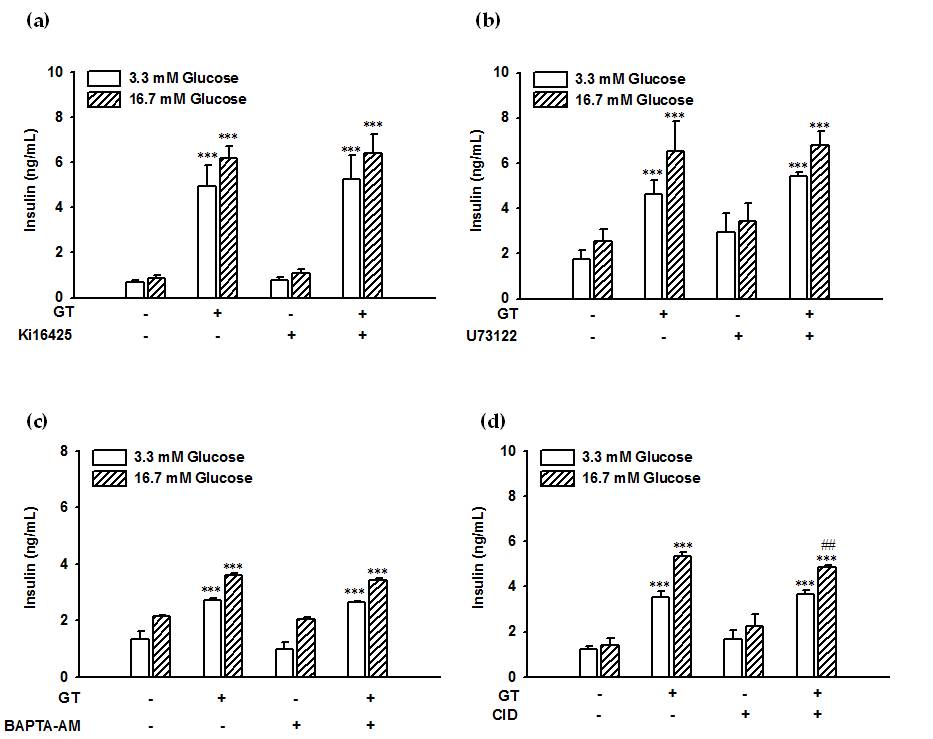


**Figure 2*.*** Effect of LPA1/3 receptor inhibitor (a), PLC inhibitor (b), calcium chelator (c) or GPR55 antagonist (d) on gintonin-induced insulin secretion from INS-1 cells. Cells were incubated either with gintonin (30 μg/mL) in KRB buffer (3.3 mM glucose or 16.7 mM glucose) for 2 h in the presence or absence of the LPA1/3 receptor inhibitor, Ki16425 (10 μM) (a),the PLC inhibitor, U73122 (5 μM) (b) , BAPTA-AM (5 μM) (c), or the GPR55 antagonist, CID1261822 (10 μM) (d). Insulin secretion was measured using an insulin ELISA kit. Data represent means ± SD (n = 4 to 6); ***p < 0.001, vs. control. GT, gintonin; CID, CID1261822.; ^##^ p < 0.01, compared to GT alone.


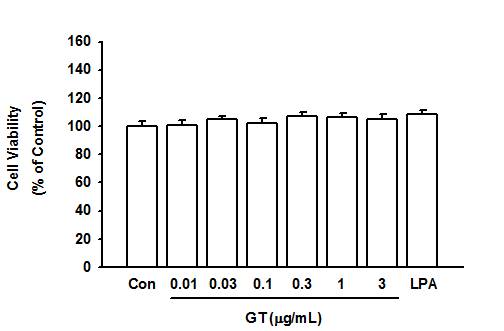


**Figure 3.** Effect of gintonin on PC-3 cell viability. Cells were incubated with the indicated concentrations of gintonin for 24 h. LPA (1 μM) was used as a positive control. Cell viability was examined by XTT-based cell viability assay. Data represent means ± SD (n = 4 to 6). Con, control; GT, gintonin.


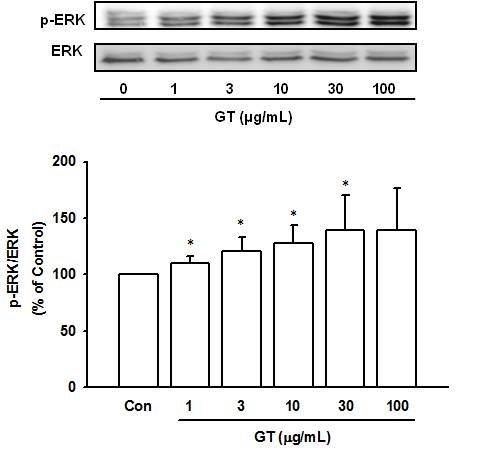


**Figure 4.** The stimulatory effect of gintonin on ERK phosphorylation in INS-1 cells. Cells were incubated with the indicated concentrations of gintonin for 10 min, lysed with lysis buffer, and subjected to immunoblotting using antibodies against ERK and p-ERK. Data represent means ± SD (n = 4 to 6); *p < 0.05, vs. control (Con). GT, gintonin.
